# Impact of Preoperative Embolization on Surgical Outcomes in Carotid Paraganglioma Resection: A Retrospective Cohort Study

**DOI:** 10.7759/cureus.86203

**Published:** 2025-06-17

**Authors:** José D Ortiz-Cisneros, Miguel Jiménez-Yarza, Carlos E Pérez-Tristán, Armando E Porras-González, Antonio Rojas-Calvillo

**Affiliations:** 1 Surgical Oncology, Instituto De Seguridad Y Servicios Sociales de Los Trabajadores Del Estado Hospital Regional, Monterrey, MEX; 2 General Surgery, Instituto De Seguridad Y Servicios Sociales de Los Trabajadores Del Estado Hospital Regional, Monterrey, MEX; 3 General Surgery, Nuevo Hospital Civil De Guadalajara “Dr. Juan I. Menchaca", Guadalajara, MEX; 4 Surgical Oncology, Instituto De Seguridad Social Del Estado De México Y Municipios, Toluca, MEX

**Keywords:** carotid, glomus, paraganglioma, preoperative embolization, tumor

## Abstract

This retrospective cohort study evaluates the impact of preoperative embolization (PE) on surgical outcomes in carotid paraganglioma resections. Carotid paragangliomas are rare, highly vascular tumors that pose significant surgical challenges due to their proximity to critical neurovascular structures. Although PE has been proposed to reduce intraoperative bleeding, operative time, and complications, its efficacy remains debated. Clinical records of 56 patients undergoing 63 resections between 2007 and 2021 at the Instituto De Seguridad Social Del Estado De México Y Municipios State Cancer Center were analyzed. Outcomes assessed included intraoperative bleeding, surgical time, hospital stay, cranial nerve and vascular complications, transfusion requirements, and late neurological sequelae. While embolization did not provide statistically significant benefits across the overall cohort, subgroup analysis revealed notable advantages in Shamblin III tumors. In this group, embolization was associated with reduced bleeding (223 mL vs. 550 mL, p=0.038), shorter operative time (146.7 min vs. 223 min, p=0.048), and decreased hospital stay (2.17 vs. 5.40 days, p=0.004). Vascular complications and late sequelae were also significantly lower in embolized Shamblin III cases (5.6% vs. 40%, p=0.043). No significant differences were observed in short-term neurological outcomes between embolized and non-embolized groups. These findings suggest that PE may offer surgical benefits in select high-risk patients, particularly those with advanced-stage tumors, while its routine use in Shamblin I and II cases appears unwarranted. Individualized decision-making based on tumor classification and surgical context remains essential to optimizing outcomes.

## Introduction

According to the Mayo Clinic, a paraganglioma is an abnormal growth originating from chromaffin cells or paraganglia, which are specialized nerve cells derived from the neural crest [1]. These cells, known as sensory chemoreceptors, have diverse functions such as detecting chemical changes in arterial blood flow and monitoring blood pressure and gas pH levels. Consequently, they play a crucial role in regulating oxygen levels and maintaining physiological homeostasis of the respiratory and cardiovascular systems [2].

Paraganglioma, also known as glomus or carotid body tumor (CBT), is a distinctive tumor situated within the adventitia of the bifurcation of the carotid artery, specifically in proximity to the carotid sinus. The carotid body is a bilateral sensory structure, typically rounded and measuring between 2 mm and 6 mm, which is an integral part of the peripheral nervous system. The initial characterization of this structure can be traced back to von Luschka in 1862 [3]. Paragangliomas located in the head and neck represent 0.6% of all tumors within this anatomical region and only 3% of all paragangliomas [4]. According to several studies, they have an estimated incidence of 1:30000, with a higher prevalence recognized among females, particularly during the fourth and fifth decades of life [5,6]. In addition, malignancy criteria are identified in 5%-7% of these tumors [7]. Although the majority are benign, they still represent a major surgical challenge due to their location and high vascularity [4].

The World Health Organization (WHO) classifies paragangliomas based on their origin into intraadrenal or extraadrenal, regardless of their secretory state. Extraadrenal paragangliomas are primarily found along the paravertebral sympathetic chains of the thorax, abdomen, and pelvis. They can originate from the sympathetic nervous system, resulting in catecholamine-secreting tumors (norepinephrine), commonly referred to as "functional tumors." On the other hand, non-secretory or "non-functional" tumors stem from the parasympathetic nervous system and are typically situated adjacent to the base of the skull, glossopharyngeal, and vagus nerve [8,9].

Surgical resection remains the primary curative treatment for CBTs. However, their intimate association with neurovascular structures renders resection complex, particularly in higher Shamblin classifications [10,11]. To reduce surgical morbidity, preoperative embolization (PE) has been employed as an adjunctive measure. The technique aims to reduce tumor vascularity and facilitate dissection. While some studies support its efficacy in decreasing operative time and intraoperative blood loss, others have found no significant benefit regarding neurologic complications, transfusion requirements, or length of hospital stay [12-16]. A recent propensity-matched analysis by Wu et al. demonstrated a significant reduction in blood loss and operative time in embolized patients, with no increase in perioperative complications [14]. Similarly, the meta-analysis by Texakalidis et al. confirmed these findings but noted no benefit in cranial nerve injury or stroke rates [17]. Conversely, Abu-Ghanem et al. concluded that PE does not confer consistent operative or postoperative advantages. These inconsistencies highlight the importance of patient selection, embolization technique, and tumor characteristics when considering PE [16].

## Materials and methods

This was a retrospective, cross-sectional, observational cohort study conducted at the Instituto De Seguridad Social Del Estado De México Y Municipios State Cancer Center in Toluca, State of Mexico. The study included patients diagnosed with carotid paraganglioma who underwent surgical resection between January 1, 2007, and July 31, 2021. All patient data were reviewed during the year 2021. The study population consisted of adult patients (over 18 years old), regardless of sex, who were scheduled for surgery and had a confirmed diagnosis of carotid paraganglioma. Eligible patients were required to have at least one month of postoperative follow-up at the Instituto De Seguridad Social Del Estado De México Y Municipios State Cancer Center and complete clinical records.

Exclusion criteria included patients who were lost to follow-up for reasons unrelated to death. Additionally, any patients whose records were incomplete or missing, whether physically or electronically, were excluded from the analysis. Each surgical procedure was considered an independent case for analytical purposes, including bilateral or metachronous tumors.

Data collection was performed using a standardized Microsoft Excel (Microsoft Corporation, Redmond, Washington, USA) spreadsheet that captured demographic, clinical, surgical, and outcome variables. To ensure consistency and reliability, all data were extracted by trained personnel under the supervision of the research team. Quality assurance was maintained by double-checking entries against original medical records.

Descriptive statistics were applied to summarize patient demographics and baseline characteristics. Continuous variables were expressed as mean ± standard deviation (SD) and compared using the Student’s t-test or the Mann-Whitney U test, depending on data distribution. Categorical variables were reported as absolute frequencies and percentages, with comparisons made using the chi-square (χ²) or Fisher’s exact test. Spearman’s correlation analysis was used to examine relationships between the degree of surgical complications and clinical variables. Multivariate analysis was performed using logistic regression and two-way analysis of variance (ANOVA) to evaluate the impact of PE on surgical outcomes, including intraoperative bleeding, surgical time, transfusion rates, and complications.

The study employed a convenience sampling strategy, with the final sample size determined by the number of patients meeting inclusion criteria during the study period. All statistical analyses were conducted using IBM SPSS Statistics software, version 23 (IBM Corp., Armonk, New York, USA).

The study protocol was reviewed and approved by the Institutional Review Board (IRB) of the Instituto De Seguridad Social Del Estado De México Y Municipios State Cancer Center, under protocol number (ISSEMYM/CEI/2021/057). All procedures were conducted in accordance with the ethical principles outlined in the General Health Law of Mexico and applicable confidentiality regulations. Due to its retrospective design, informed consent was waived; however, patient anonymity was preserved throughout the study.

Potential sources of bias

Given the retrospective design, selection bias may have influenced the decision to perform embolization. Factors such as tumor vascularity, surgeon preference, and institutional protocol likely contributed to treatment allocation. These unmeasured confounders were not uniformly documented and represent a limitation of this study.

## Results

**Figure 1:**
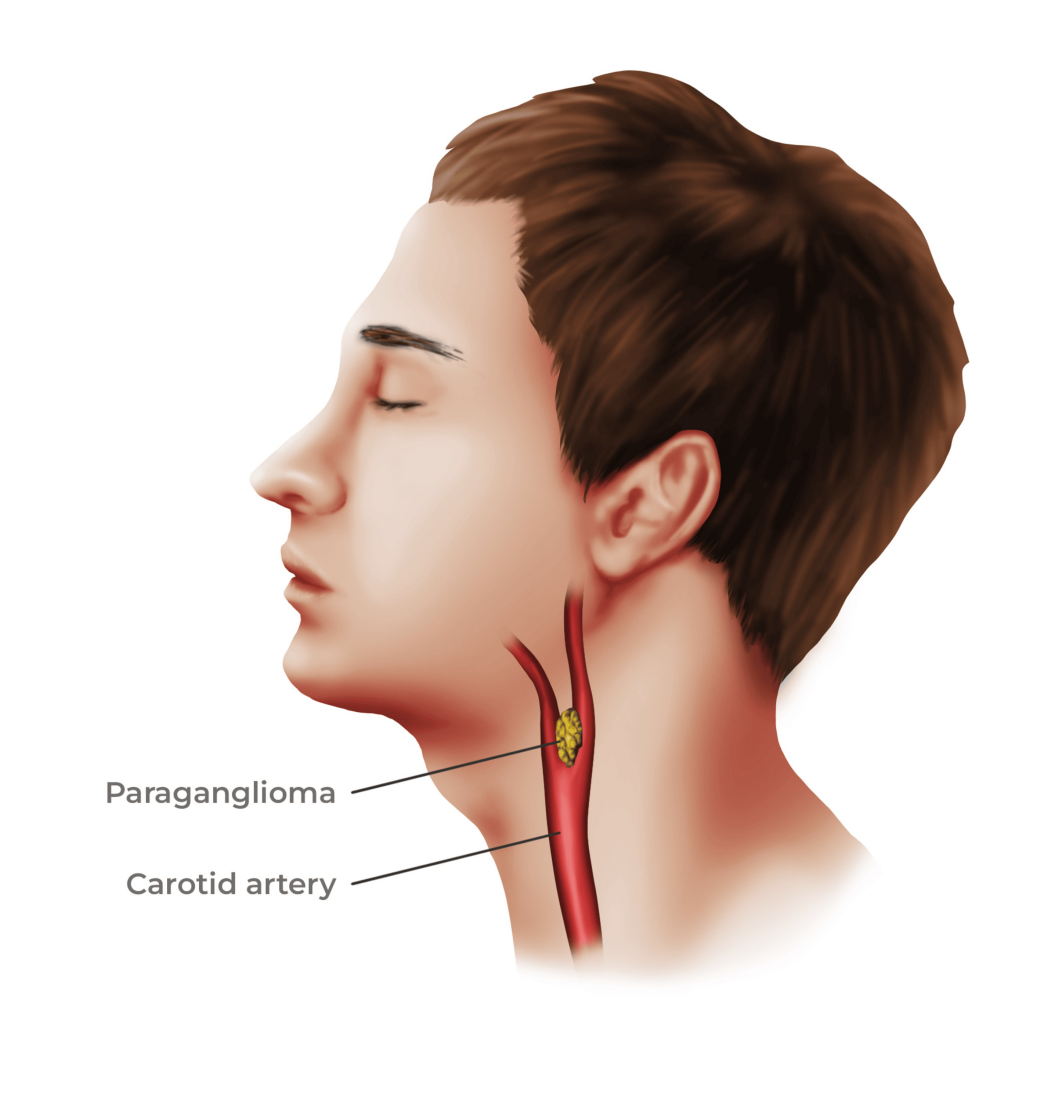
Carotid paraganglioma Image credits: Miguel Jiménez-Yarza

From January 2007 to July 31, 2021, a comprehensive analysis encompassed 61 registered patients who underwent 68 carotid paraganglioma resections. These procedures involved both embolization and non-embolization techniques before resection of the carotid paraganglioma (Figure 1).

Five patients were excluded due to incomplete clinical records, either in electronic or physical form, hindering data collection aligned with the study's objectives. Consequently, 63 paragangliomas in 56 patients met the specified inclusion criteria.

For analytical purposes, each paraganglioma was treated as an independent patient, given the distinct nature of each procedure. The surgeries occurred as separate events, with selective embolization allowing for individualized analysis for each resection.

In the cohort of 56 patients under scrutiny, the average age was 52.6±10.04 years, ranging from 31 to 72 years. In cases of bilateral presentation, the age at diagnosis was considered for synchronous instances, while for metachronous occurrences, the age at diagnosis of the first carotid paraganglioma was utilized. It is worth noting that one patient had a prior diagnosis of vagal paraganglioma treated elsewhere, necessitating the use of the age at diagnosis for the carotid paraganglioma due to the unavailability of the age at the time of the initial diagnosis of the vagal paraganglioma.

Of the analyzed paraganglioma population (n=56) 52 were female and four male (Table 1). As for the pre-surgical diagnosis, 12 (19.0%) were classified as Shamblin I, 28 (44.4%) Shamblin II, and 23 (36.5%) Shamblin III were observed (Table 1). Regarding laterality, 34 paragangliomas (53.97%) are located on the left side and 29 (46.03%) on the right side (Table 1). Regarding bilaterality, it was detected in nine patients, comprising 16.07% of the total study population. It is important to highlight that among these cases, eight patients underwent resection with at least a six-month interval between their surgeries. One patient had a history of contralateral paraganglioma resected in another medical facility, while another patient had a previous diagnosis of vagal paraganglioma. Therefore, the latter diagnosis was excluded from the analysis. Despite sharing common pathophysiology and genetic backgrounds, this study's focus lies specifically on describing carotid glomus tumors (Table 1).

**Table 1:** Summary of patient demographics and tumor characteristics This table summarizes the key demographic and clinical characteristics of the 56 patients included in the study. Variables include gender distribution, Shamblin classification of carotid paragangliomas, tumor laterality, presence of bilaterality or multifocality, and whether PE was performed. Values are expressed as percentages of the total patient population. PE: preoperative embolization

Variable	Frequency, n (%)
Gender - female	52 (92.96%)
Gender - male	4 (7.14%)
Shamblin I	12 (19.05%)
Shamblin II	28 (44.44%)
Shamblin III	23 (36.51%)
Laterality - left	34 (53.97%)
Laterality - right	29 (46.03%)
Bilaterality - present	9 (16.07%)
Bilaterality - absent	47 (83.93%)
Multifocality - present	2 (3.17%)
Multifocality - absent	61 (96.83%)
Embolization - performed	26 (41.27%)
Embolization - not performed	37 (58.73%)

Multifocality was identified in only two of the resections performed, constituting 3.17% of the cases. It is essential to note that multifragmented tumors were excluded from this analysis, and only those defined as independent tumors in the histopathological report were considered (Table 1). PE was conducted in 26 paragangliomas (41.27%), whereas 37 (58.73%) were resected without embolization (Table 1).

**Figure 2:**
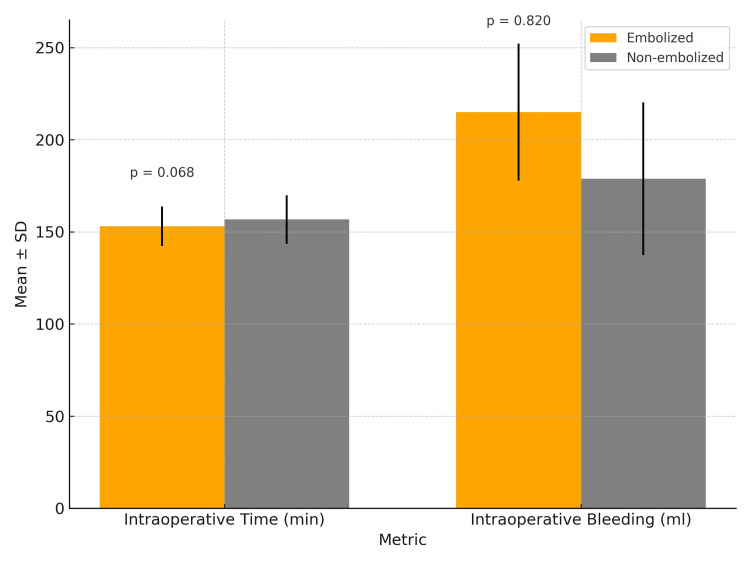
Comparison of intraoperative time and bleeding in embolized vs. non-embolized carotid paraganglioma resections

The average intraoperative time recorded was 155.22±70.08 min, with bleeding averaging 193.81±127.36 mL (Figure 2).

Vascular complications occurred in five of the resections, representing a rate of 7.9%. Among these cases, three (4.76%) involved tears necessitating immediate vascular repair with raffia, while one case (1.59%) required major vascular ligation (common carotid artery). It is noteworthy that this particular patient experienced contralateral body hemiplegia; although not categorized as a neurological complication due to the primary focus on describing cranial parenchymal lesions, it remains important to acknowledge this complication. Additionally, there was one instance (1.59%) of postoperative bleeding leading to hematoma formation, necessitating reintervention with cervical exploration.

In the analysis of neurological complications within the 30-day postoperative period, a complication rate related to cranial pairs of 23.8% was observed. This breakdown included 4.76% attributed to the vagus nerve (CN X) resulting in dysphagia, 3.17% linked to the upper laryngeal nerve causing dysphonia, 7.94% associated with the hypoglossal nerve (CN XII) presenting as tongue deviation or dysarthria, and 6.35% involving the facial nerve (CN VII), particularly its marginal branch, resulting in corner-of-the-mouth deviation post-resection.

It is noteworthy that in three paraganglioma resections, neurological conditions manifested concurrently in more than one cranial nerve. Specifically, one patient experienced conditions in four cranial nerves: CN VII in its marginal branch, CN X (vagus nerve) main branch, CN X (superior laryngeal nerve), and CN XII (hypoglossal nerve). Another patient exhibited sequelae in two cranial pairs: dysphonia due to upper laryngeal nerve (CN X) involvement and dysphagia due to esophageal branches of the vagus nerve (CN X) impairment. Additionally, a patient displayed corner-of-the-mouth deviation and tongue deviation due to affection of the marginal branch (CN VII) and the hypoglossal nerve (CN XII), respectively.

Regarding late neurological sequelae (occurring >12 months post-resection), nine patients did not meet the follow-up duration exceeding one year, precluding their inclusion in this analysis. Notably, none of these cases were bilateral, resulting in 54 resections being considered as the total sample size. Among these cases, sequelae were observed in 5.6% (n=3) of patients. Specifically, one patient (1.85%) continued to experience dysphonia, another (1.85%) exhibited corner-of-the-mouth deviation due to involvement of the marginal branch of the facial nerve, and one (1.85%) presented with tongue deviation, a consequence of hypoglossal nerve injury.

The utilization of hemoderivatives was also scrutinized, revealing that out of the 63 resections, only two cases required transfusions, constituting a rate of 3.17%.

Furthermore, a comparison of surgical time and bleeding was conducted between resections with and without PE. The analysis revealed an average bleeding volume of 215.0±37.2 mL versus 178.92±41.4 mL (p=0.820). Notably, a shorter surgical duration was observed in the group of patients with PE compared to those without embolization (153.0±10.7 min vs. 156.7±13.1 min), although this disparity did not reach statistical significance (p=0.068).

An analysis was conducted using the Chi-square (χ²) test to compare the two previously described groups based on vascular complications. It was observed that among the total paragangliomas without prior embolization (n=37), a complication rate of 8.1% was noted. In contrast, for the group of paragangliomas with prior embolization (n=26), the complication rate was 7.7%. These findings did not reveal any statistically significant differences between the two groups (p=0.952).

Similarly, both groups were compared based on short-term neurological complications. It was observed that for the group without embolization (n=37), the rate of neurological complications was 16.2%, while in the group with embolization (n=26), it was 15.4%. No statistically significant difference was observed between the two groups (p=0.929).

Late neurological complications were assessed and compared between the groups under study, excluding nine patients who did not comply with the follow-up duration exceeding one year (n=54), none of whom were bilateral cases. In the group without embolization, a rate of 6.5% was observed, whereas in the embolization group, it was 4.3%. No statistically significant differences were noted between the two groups.

Regarding the use of hemoderivative transfusion, it was found that it only occurred in the group without prior embolization, with a rate of 5.4% among the total paragangliomas studied. However, this difference did not reach statistical significance (p=0.228) (Table 2).

**Table 2:** Summary of postoperative complications and outcomes in carotid paraganglioma resections

Outcome	n (%)
Vascular complications - total	5 (7.9%)
Tear requiring repair (raffia)	3 (4.76%)
Major vascular ligation (hemiplegia)	1 (1.59%)
Hematoma with reintervention	1 (1.59%)
Neurological complications ≤30 days	15 (23.8%)
CN X - vagus (dysphagia)	3 (4.76%)
CN X - superior laryngeal (dysphonia)	2 (3.17%)
CN XII - hypoglossal (tongue deviation)	5 (7.94%)
CN VII - marginal branch (mouth deviation)	4 (6.35%)
Post-embolization tinnitus	1 (1.59%)
Late neurological sequelae >12 months	3 (5.6%)
CN XII - hypoglossal	1 (1.85%)
CN VII - marginal branch	1 (1.85%)
CN X - laryngeal (dysphonia)	1 (1.85%)
Transfusions required	2 (3.17%)
Vascular complications - with embolization	2/26 (7.7%)
Vascular complications - without embolization	3/37 (8.1%)

The duration of hospital stay in both study groups was evaluated and compared. It was observed that among the total paragangliomas with prior embolization, there was a longer average time in days (2.19±0.56) compared to the non-embolization group (1.97±2.4), with a statistically significant difference (p=0.02).

The bleeding, intraoperative time, and hospital stay were compared according to the Shamblin classification, specifically in category II for both study groups. Among the eight paragangliomas with PE, the average bleeding was (173.75±26.7 mL) compared to (141.75±69.5 mL) for the group without prior embolization, with no statistically significant difference observed. Similarly, when evaluating the average surgical time, it was found that the group with embolization had an average time of (167.5±20.4) min compared to (167.5±17.2) min for the group without embolization, respectively, showing no significant difference. Finally, the duration of hospital stay was assessed, revealing an average of 2.25 days for the embolization group and 1.65 days for the non-embolization group, without statistical significance.

An analysis of vascular and neurological complications, as well as late sequelae, was conducted in patients with Shamblin II classification in the two study groups. In the group without prior embolization, 95% of patients did not experience any vascular complications, while only 5% encountered such events. Regarding neurological complications, a rate of 10% was observed in the group without embolization, compared to 12.5% in the group with embolization, with no significant differences between the two groups. Similarly, there were no discernible differences in the occurrence of late sequelae between the two groups.

It was also considered to conduct an analysis for these same complications based on the subdivision by the size of the Shamblin II paragangliomas (<40 mm and >40 mm), as proposed in the modification of the original classification. However, it was not feasible to perform a statistical test for the disparity between the groups due to the limited sample size for paragangliomas classified as Shamblin II and greater than 40 mm without embolization.

Finally, bleeding, intraoperative time, and hospital stay were compared according to Shamblin III classification in the two study groups. Among the 18 patients with PE, the average bleeding was (223±44.6 mL) compared to (550±232 mL) for the group without embolization, showing a statistically significant difference (p=0.038). Similarly, when evaluating the average surgical time, it was found that the group with embolization had an average time of (146.7±12.63 min) compared to (223.0±49.6 min) for the group without embolization, with statistical significance (p=0.048). Finally, the duration of hospital stay was assessed, revealing an average of 2.17 days for the embolization group compared to 5.40 days for the non-embolized group, demonstrating statistical significance (p=0.004) (Table 3).

**Table 3:** Bleeding analysis, surgical time, and hospital stay in patients with Shamblin III Median values of bleeding, surgical time, and hospital stay in Shamblin III tumors with and without PE. P-values were calculated using the Chi-square test for group comparisons. PE: preoperative embolization

Variable	Embolization	N	Median	P-value (Chi-square)
Bleeding (mL)	Absent	5	550	0.038
Bleeding (mL)	Present	18	223	0.038
Surgical time (min)	Absent	5	233.3	0.048
Surgical time (min)	Present	18	146.67	0.048
Days of hospitalization	Absent	18	5.4	0.004
Days of hospitalization	Present	10	2.17	0.004

In the multivariate analysis of complications (vascular and neurological), as well as late sequelae in paragangliomas classified as Shamblin III, it was observed that vascular complications occurred in 40% of patients in the non-embolized group, whereas a 5.6% complication rate was observed in those who underwent prior embolization before resection. The difference between the two groups was found to be significant (p=0.043). Regarding neurological complications, no significant differences were observed between the two groups (p=0.263). However, when evaluating late sequelae, a significant difference was found in favor of the group with prior embolization compared to the group without embolization (5.6% vs. 40%, p=0.043) (Table 4).

**Table 4:** Multivariate analysis for embolization Multivariate analysis using ANOVA to assess the impact of embolization on vascular and neurological complications, transfusion requirements, bleeding, and surgical time. Results are presented as OR with 95% CI. OR: odds ratios; CI: confidence intervals; ANOVA: analysis of variance

Outcome	OR (95% CI)	P-value (ANOVA)
Vascular complications (present vs. absent)	0.944 (0.146-6.091)	0.952
Neurological complications (present vs. absent)	0.939 (2.37-3.727)	0.926
Transfusion required (present vs. absent)	0.574 (0.462-0.712)	0.228
Bleeding (continuous variable)	1.58 (0.59-1.10)	0.11
Surgical time (continuous variable)	1.34 (0.366-716)	0.716

A multivariate analysis was conducted utilizing logistic regression and two-way ANOVA tests to examine the influence of embolization on surgical variables, vascular complications, and short-term neurological outcomes. The Shamblin III classification emerged as the sole independent factor significantly associated with vascular complications (p=0.048) (Tables 5, 6).

**Table 5:** Multivariate analysis of vascular complications Analysis of vascular complications using ANOVA, comparing patient subgroups by Shamblin classification, laterality, transfusion status, embolization, bleeding, and surgical time. Values are shown as OR with 95% CI. OR: odds ratios; CI: confidence intervals; ANOVA: analysis of variance

Variable	Yes (n=5)	No (n=58)	OR (95% CI)	P-value (ANOVA)
Shamblin I	0 (0%)	12 (100%)	0.90 (0.82-0.98)	0.258
Shamblin II	2 (7.1%)	26 (92.9%)	1.11 (0.92-1.31)	0.835
Shamblin III	3 (13%)	20 (87%)	3.07 (0.47-20.04)	0.048
Right side	4 (13.8%)	25 (86.2%)	1.89 (0.20-1.80)	0.112
Left side	1 (2.9%)	33 (97.1%)	4.69 (0.55-39.5)	0.262
Transfusion	1 (50%)	1 (50%)	1.89 (0.45-7.48)	0.364
Without transfusion	4 (6.6%)	57 (93.4%)	0.53 (0.13-2.14)	0.25
Embolization	2 (7.7%)	24 (92.3%)	1.05 (0.86-5.87)	0.952
Without embolization	3 (8.1%)	34 (91.9%)	0.99 (0.86-1.15)	0.927
Bleeding (mL)	361	179	1.98 (0.198-1.84)	0.843
Surgical time (min)	224	149	0.66 (0.43-1.06)	0.665

**Table 6:** Multivariate analysis of short-term neurological complications Multivariate ANOVA analysis of neurological complications by Shamblin classification, laterality, transfusion status, embolization, bleeding, and surgical time. Data are shown as OR with 95% CI. OR: odds ratios; CI: confidence intervals; ANOVA: analysis of variance

Variable	Yes (n=10)	No (n=53)	OR (95% CI)	P-value (ANOVA)
Shamblin I	2 (16.7%)	10 (83.3%)	1.07 (1.97-5.85)	0.933
Shamblin II	3 (10.7%)	25 (89.3%)	0.480 (0.11-2.05)	0.316
Shamblin III	5 (21.7%)	18 (78.3%)	1.94 (0.497-7.60)	0.784
Right side	5 (17.2%)	24 (82.8%)	0.97 (0.78-1.20)	0.526
Left side	5 (14.7%)	29 (85.3%)	1.72 (0.37-3.65)	0.18
Transfusion	9 (14.8%)	52 (85.2%)	0.395 (0.65-1.33)	0.294
Without transfusion	1 (50%)	1 (50%)	1.70 (0.42-6.84)	0.928
Embolization	4 (15.4%)	22 (84.6%)	1.05 (0.330-3.36)	0.608
Without embolization	6 (16.2%)	31 (83.8%)	0.99 (0.79-1.23)	0.602
Bleeding (mL)	241	184	1.98 (0.198-1.84)	0.843
Surgical time (min)	159	154	1.98 (0.84-2.38)	0.665

**Figure 3:**
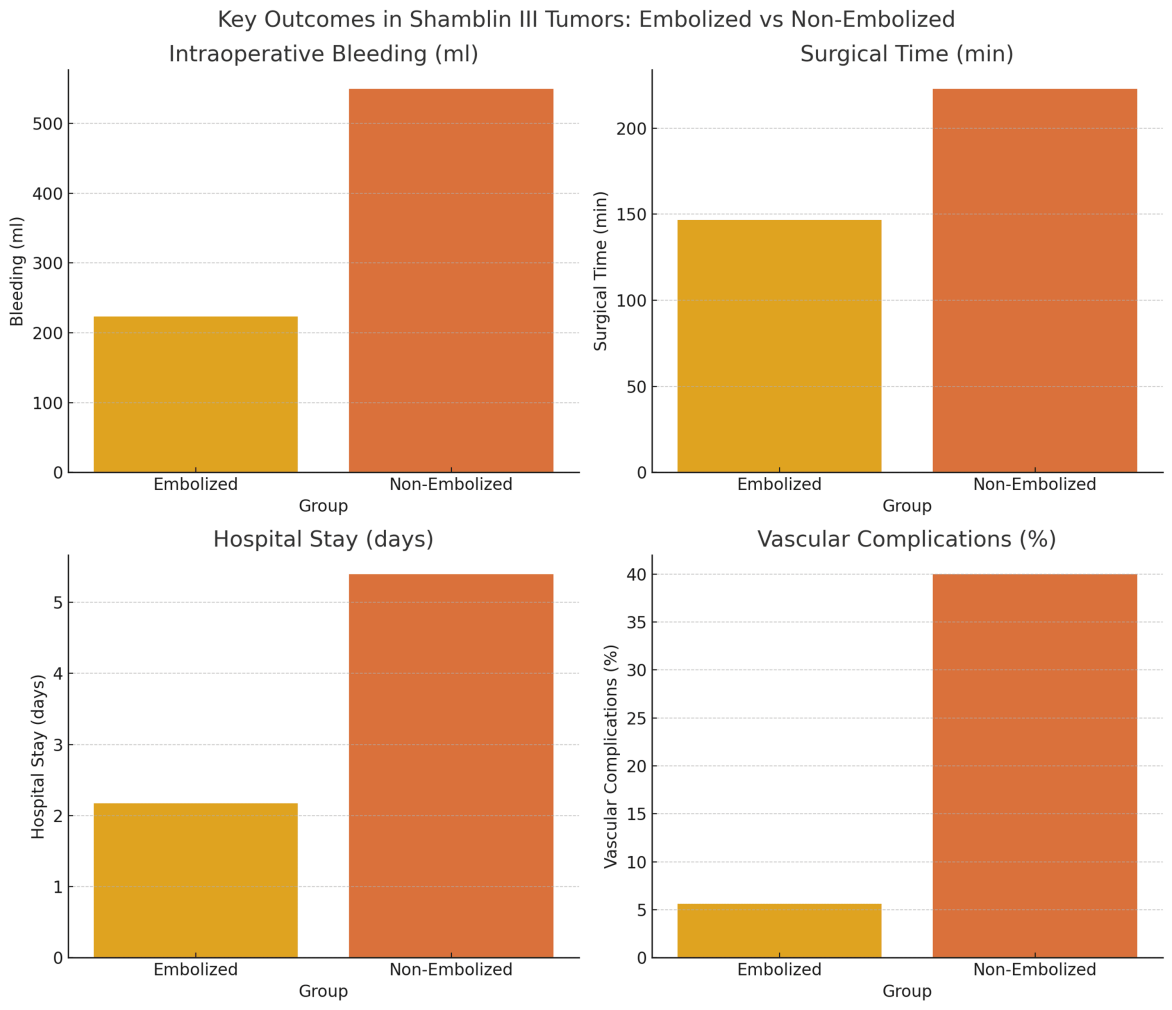
Comparison of key surgical outcomes in Shamblin III tumors with and without PE Embolization was associated with significant reductions in intraoperative bleeding, surgical time, hospital stay, and vascular complications. Data represent group means or percentages. PE: preoperative embolization

Figure 3 shows a summary of the most relevant findings of the study regarding Shamblin III tumors.

## Discussion

The primary objective of this research was to assess the potential surgical benefits associated with PE in patients diagnosed with carotid paraganglioma undergoing resection. The study revealed that the average age of the participants was 52.61±10.04 years, ranging from a minimum of 31 years to a maximum of 72 years. Among the studied population (n=56), 92.9% of paragangliomas (n=52) occurred in female patients, with only 7.1% (n=4) observed in male patients. While the gender distribution in this study is relatively higher among women compared to other reports in the literature, this aligns with the documented predominance of female cases in similar studies [8].

Our findings showed a higher average bleeding volume in the embolized group (215.0±37.2 mL) compared to the non-embolized group (178.92±41.4 mL), without statistical significance. The non-embolized group had a larger proportion of Shamblin I tumors, which may have skewed bleeding outcomes. Similar variability has been documented in the literature, with some studies reporting significantly reduced bleeding with embolization while others reported no difference [10,14,16,17].

Surgical time was slightly shorter in embolized cases (153.0±10.7 min vs. 156.7±13.1 min), also without statistical significance. Previous studies, including Jackson et al. and Wu et al., found embolization associated with reduced operative time [13,14].

Regarding complications, vascular injury occurred in 7.9% of patients. No significant difference was observed between embolized and non-embolized groups, consistent with earlier reports [10,16]. Yildirim et al. suggested that embolization is only protective when devascularization exceeds 90%, with lower vascular complication rates in that subgroup [18].

Hospital stay was longer in the embolized group (2.19±0.56 days vs. 1.97±2.4 days, p=0.02), attributable to our protocol of preoperative admission for embolization. No differences were noted postoperatively. Similar institutional variations have been described in other reports [10,15].

Subgroup analysis by Shamblin classification revealed no benefit in Shamblin II tumors. However, Shamblin III patients demonstrated significantly lower blood loss, operative time, and vascular complication rates in the embolized group. These results support PE in advanced tumors, as previously noted by Texakalidis et al. and Yildirim et al. [17,18].

The work by Kim et al. introduced DTBOS and tumor volume as additional predictors of surgical risk. When combined with Shamblin grade, these variables improved the prediction of blood loss and cranial nerve injury. This reinforces the need for individualized embolization strategies [19].

This study's limitations include its retrospective nature, potential selection bias, and the absence of long-term follow-up. It is also important to acknowledge that embolization outcomes may be influenced by procedural variables such as the degree of devascularization achieved, the type of embolic agent used (e.g., polyvinyl alcohol particles vs. coils), and the experience of the interventional radiologist. These factors were not controlled for in this study due to its design, yet they may significantly impact surgical outcomes and highlight the need for standardized reporting in future analyses. Despite these limitations, our results contribute real-world data supporting the selective use of embolization, especially in high-risk tumors.

PE may simplify CBT resection, particularly in Shamblin III tumors, by reducing bleeding and operative time. However, its benefits are not uniform across all cases. Careful patient selection based on tumor size, vascularity, and proximity to critical structures is essential for optimizing outcomes.

## Conclusions

Following the analysis of our results, it is evident that the benefits of PE in carotid paraganglioma resection are closely tied to the Shamblin classification, with the most substantial advantages observed in Shamblin III tumors. In this group, embolization significantly reduced intraoperative bleeding, surgical time, hospital stay, and vascular complications, supporting its routine use in complex cases. Conversely, embolization did not provide measurable benefits in Shamblin I tumors and should not be routinely performed, as the risks may outweigh the potential gains. For Shamblin II tumors, an individualized approach is recommended, taking into account tumor characteristics, patient factors, and surgeon expertise. Importantly, no significant differences were found in cranial nerve-related complications across groups. These findings support a selective, classification-based strategy for embolization. However, it is essential to acknowledge the limitations inherent in this retrospective design, including potential confounders such as surgeon experience and procedural complexity. While a prospective, randomized trial would offer more definitive guidance, the rarity of this condition poses practical challenges. Future studies should focus on prospective multicenter trials incorporating volumetric tumor analysis and vascular imaging data to refine embolization decision-making protocols, especially in intermediate-risk tumors. Further research, especially exploring tumor size subgroups within Shamblin II, is warranted to refine embolization criteria and optimize surgical outcomes.
